# Factors Influencing on the Aneurysm Sac Shrinkage after Endovascular Abdominal Aortic Aneurysm Repair by the Analysis of the Patients with the Aneurysm Sac Shrinkage and Expansion

**DOI:** 10.3400/avd.avd.oa.23-00065

**Published:** 2023-09-28

**Authors:** Atsushi Aoki, Kazuto Maruta, Tomoaki Masuda, Tadashi Omoto

**Affiliations:** 1 Department of Cardiovascular Surgery, Showa University, Tokyo, Japan

**Keywords:** endovascular abdominal aortic aneurysm repair, coil embolization, tranexamic acid, antifibrinolytic therapy, aneurysm sac shrinkage

## Abstract

**Objectives:** The aneurysmal sac shrinkage has been reported as the strong predictor of favorable long-term outcome after endovascular aneurysm repair (EVAR). We evaluated the effects of perioperative and intraoperative factors on the aneurysm sac shrinkage.

**Methods:** EVAR was performed for 296 patients during August 2009–December 2021. Nine patients with type Ia, Ib, or III; 69 patients with the sac diameter change less than 5 mm; and five patients with sac re-expansion after shrunk more than 5 mm were excluded. Thus, patients with sac shrinkage 5 mm or more (79 patients, shrinkage group) and with sac expansion 5 mm or more (18 patients) were included in this study. Antifibrinolytic therapy with tranexamic acid (TXA) 1500 mg/day for 6 months after EVAR was introduced in March 2013 and patent aortic side branches were coil embolized during EVAR since July 2015. Patients’ background and patent aortic side branches at the end of EVAR were evaluated.

**Results:** Univariate analysis for comparison between patients with sac shrinkage and sac expansion revealed that males (82.3% vs. 55.6%, p = 0.021), without antiplatelet therapy (40.5% vs. 66.7%, p = 0.044) and TXA (79.8% vs. 38.9%, p <0.001), were significantly associated with sac shrinkage. By multivariate analysis, the odds ratio of sac shrinkage was 11.7 for males, 0.1 for the patients on antiplatelet therapy, and 6.5 for the patient who received TXA. The patients with patent inferior mesenteric artery (IMA) were less in the shrinkage group (20.3% vs. 77.8%, p <0.001) and with two or less patent lumbar arteries (LAs) were more in the shrinkage group (82.3% vs. 33.3%, p < 0.001). The odd ratio of sac shrinkage was 7.8 for occluded IMA and 3.9 for two or less patent LAs.

**Conclusion:** The possibility of sac shrinkage would be high for the patient with occluded IMA and two or less patent LA at the end of EVAR, and that patient received TXA after EVAR.

(This is a translation of *Jpn J Vasc Surg* 2022; **31**: 291–297.)

## Introduction

The advantage of endovascular aneurysm repair (EVAR) of abdominal aortic aneurysm (AAA) is less invasiveness; however, approximately 25% of patients experience aneurysm expansion in a report by the Japanese Committee for Stent Graft Management,^1)^ and unfavorable long-term outcome is the disadvantage of EVAR. Aneurysm sac shrinkage after EVAR suggests that the aneurysm has been completely excluded from blood flow and is a strong predictor of a good long-term prognosis.^2)^ In the present study, we examined factors that affect aneurysm shrinkage following EVAR.

## Participants and Methods

**Figure 1:**
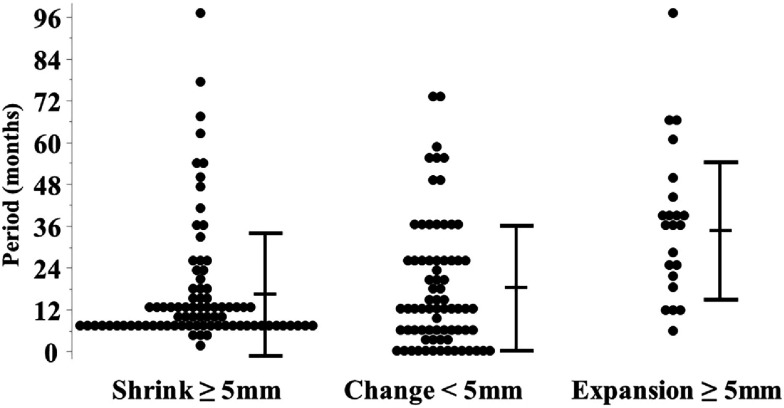
Fig. 1 Period until sac shrinkage or expansion 5 mm detection or follow-up period for patients with sac diameter change less than 5 mm. The longest follow-up period for patients with the sac diameter change less than 5 mm was shorter than the longest period until shrinkage or expansion 5 mm detection.

During the period from August 2009 to December 2021, 430 patients underwent the surgery for abdominal aortic disease at our hospital, and surgery for true AAA (including concomitant iliac artery aneurysm) was performed in 296 patients, EVAR in 214 patients, and artificial graft replacement in 82 patients. Among EVAR patients, 34 patients without preoperative and/or postoperative contrast-enhanced computed tomography (CT) data were excluded, and thus, the data of 180 patients were evaluated. First, high-pressure endoleak (HP-EL), such as type Ia, Ib, and III are considered risk factors for aneurysm rupture regardless of the aneurysm sac expansion or shrinkage. Therefore, nine patients were excluded in whom contrast enhancement was observed from the proximal or distal neck, or stentgraft junction into the aneurysm sac during the follow-up period. Next, we extracted patients with an aneurysm shrinkage or expansion of 5 mm or more and evaluated the period up to confirmation of shrinkage or expansion after EVAR. As a result, there were 84 patients with shrinkage, in whom the period until shrinkage was 1.8–97.0 months with average 15.5 months, and 18 patients with expansion, in whom the period until expansion was 5.9–97.2 months with average 37.5 months, and some patients were found to have shrinkage or expansion after 8 years or more had elapsed. In 69 patients with a change in aortic diameter of less than 5 mm at the time of the final follow-up, the follow-up observation period was less than 8 years, with a maximum of 73.2 months. The possibility that the aneurysm would shrink or expand by 5 mm or more if the follow-up observation period were longer cannot be ruled out. Therefore, these 69 patients were excluded from the study (**Fig. 1**). In the 18 patients with an expansion of 5 mm or more, there were no patients showing shrinkage after the expansion of 5 mm or more was confirmed, and these patients were defined as the expansion group. On the other hand, among 84 patients with confirmed shrinkage of 5 mm or more, re-expansion following shrinkage was observed in five patients. Among these five patients, type II endoleak (T2-EL) was observed on contrast-enhanced CT 6 months after EVAR in two patients. However, the aortic diameter shrank by 5 mm or more and then re-expanded 2 years and 6 years later. In three patients, no definite endoleak was observed, shrinkage of 5 mm or more was observed 1 year after surgery, and aneurysm sac expanded more than 5 mm.

In the 79 patients excluding these five patients, no re-expansion was observed during the follow-up period after confirmation of shrinkage of 5 mm or more (42% in <1 year, 30% in 1–3 years, and 28% in ≥3 years), and these 79 patients constituted the shrinkage group. In the present study, the expansion and shrinkage groups were included.

EVAR was performed under general anesthesia after cutting down the unilateral or bilateral femoral arteries. The procedure was performed in the angiographic suite until December 2012, in the operating room during January 2013 to June 2015 using a mobile digital subtraction angiography device (Siemens Arcadis; Siemens, Erlangen, Germany), and in the hybrid operating room equipped with a Siemens Artis Zeego (Siemens) from July 2015 onward. From March 2013, we prescribed oral tranexamic acid (TXA) 1500 mg per day from the morning after EVAR and TXA was continued for 6 months. From July 2015, we have attempted coil embolization using Interlock (Boston Scientific, Marlborough, MA, USA) for patent inferior mesenteric arteries (IMAs) and patent lumbar arteries (LAs) with an internal diameter of 2 mm or more, or with an internal diameter of less than 2 mm when there are four or more patent LAs. Coil embolization was deemed successful when at least one Interlock could be placed in the aortic branch. Aortic branches supposed to be patent at EVAR completion (patent aortic branches) included aortic branches whose origin was not covered with stent graft because the aortic diameter was larger than the diameter of stent graft deployed, and coil embolization was not attempted or was unsuccessful.

For follow-up schedule, contrast-enhanced CT was performed 1 week after surgery and 6 months after if renal function was normal. Thereafter, as a rule, plain CT was performed, but if the aneurysm sac tended to expand, contrast-enhanced CT was performed.

Statistical analysis was performed using JMP Pro 16 (SAS Inc., Cary, NC, USA). A chi-square or Fisher’s exact test was applied for categorical variables, and a Wilcoxon test was used for continuous variables. Cutoff values of predictive factors were estimated by receiver operating characteristic (ROC) analysis using the Youden’s index. Multivariate analysis was performed using a multiple logistic analysis, and a p value of less than 0.05 was considered as significant.

The present study was conducted based on the Declaration of Helsinki and with the approval of the Showa University Research Ethics Review Board (reception number 3125).

## Results

In the univariate analysis of the patient background, the shrinkage group was predominantly male, with significantly fewer patients taking some kind of antiplatelet agent and many patients taking postoperative oral TXA (**Table 1**). Upon performing multivariate analysis of factors with a p value of <0.05, male and oral TXA were significant shrinkage factors, and antiplatelet therapy was an inhibitor of aneurysm shrinkage (**Table 2**).

**Table 1:** Univariate analysis of patient background, operative factors, patent aortic side branch at the end of EVAR, and type II endoleak.

	Shrinkage (n = 79)	Expansion (n = 18)	p value
Patient back ground			
Age (years)	75.7 ± 8.0	77.9 ± 5.2	0.2893
Male	65 (82.3%)	10 (55.6%)	0.0210
Height (cm)	163.2 ± 8.7	158.7 ± 9.9	0.0834
Weight (kg)	63.8 ± 12.8	60.4 ± 12.3	0.5189
Body surface area (m^2^)	1.68 ± 0.19	1.61 ± 0.20	0.2498
Body mass index	23.8 ± 3.5	23.8 ± 3.4	0.5777
Preoperation medical therapy			
Anticoagulation therapy	8 (10.1%)	2 (11.1%)	0.2835
Antiplatelet therapy			
Any	32 (40.5%)	12 (66.7%)	0.0437
DAPT or other than aspirin	25 (31.7%)	7 (38.9%)	0.5593
Post EVAR TXA	63 (79.8%)	7 (38.9%)	0.0009
Operative factors			
Internal iliac artery			
Coil embolization	10 (12.7%)	3 (16.7%)	
Iliac bifurcated endoprosthesis	3 (3.8%)	0	0.8440
Implanted device			
Endurant	35 (44.3%)	7 (38.9%)	
Excluder	27 (34.2%)	7 (38.9%)	
Powerlink/AFX	11 (13.9%)	2 (11.1%)	
Zenith	6 (8.8%)	2 (11.1%)	0.8921
Patent aortic side branch at the end of EVAR			
IMA	16 (20.3%)	14 (77.8%)	<0.0001
Number of LAs	1.51 ± 1.48	3.28 ± 1.96	0.0006
≤2	65 (82.3%)	6 (33.3%)	<0.0001
Type II endoleak after EVAR			
1 week	7/76 (9.2%)	16 (88.9%)	<0.0001
6 months	2/48 (4.2%)	8/12 (66.7%)	<0.0001
1 year	0/9 (0%)	6/6 (100%)	0.0002
2 years or more	0/10 (0%)	11/13 (84.6%)	<0.0001
At any time	8 (10.1%)	16 (88.9%)	<0.0001

Gender, antiplatelet therapy, and antifibrinolytic therapy with TXA differed significantly between 2 groups in the patient background. The frequency of patent IMA was significantly less in the shrinkage group. The number of patent LAs was significantly less and frequency of patients with 2 or less patent LAs was significantly less in the shrinkage group. The frequency of type II endoleak was significantly less throughout the follow-up period. EVAR: endovascular aneurysm repair; DAPT: dual antiplatelet therapy; TXA: tranexamic acid; IMA: inferior mesenteric artery; LA: lumbar artery

**Table 2:** Multiple logistic regression analysis of patient backgrounds and patent aortic side branch at the end of EVAR for aneurysm sac shrinkage.

Risk factor	Estimated value	Standard error	Wald chi-square	Odds ratio	95% confidence interval	p value
Patient background						
Male gender	1.2312	0.3251	7.9051	11.73	2.443–87.92	0.0016
Antiplatelet therapy	−1.1400	0.4295	7.0449	0.102	0.014–0.457	0.0018
TXA	0.9395	0.3116	9.0894	6.547	1.990–23.62	0.0019
Patent aortic side branch at the end of EVAR procedure						
Occluded IMA	1.0246	0.3448	8.2907	7.762	2.103–33.20	0.0030
Patent LA ≤2	0.6835	0.3299	4.2914	3.903	1.079–14.76	0.0383

Male gender, antiplatelet therapy, and TXA were significant factors for aneurysm sac shrinkage and odd ratio of sac shrinkage was 11.7 for male gender, 0.1 for antiplatelet therapy, and 6.5 for antifibrinolytic therapy with TXA. When IMA was occluded and patent LA was 2 or less, the possibility of sac shrinkage would be significantly high, as odd ratio was 7.8 for occluded IMA and was 3.9 for patent LA 2 or less. EVAR: endovascular aneurysm repair; TXA: tranexamic acid; IMA: inferior mesenteric artery; LA: lumbar artery

In surgical factors, we found no significant difference in the frequency of coil embolization of the internal iliac artery and iliac bifurcation endoprosthesis placement, and in the implanted device.

**Figure 2:**
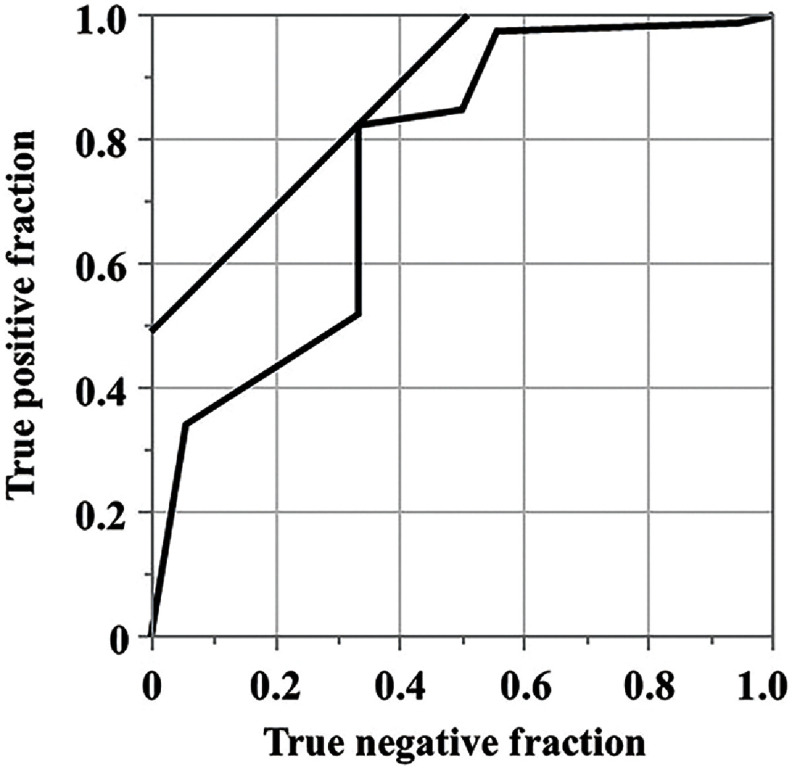
Fig. 2 ROC analysis for aneurysm sac shrink/expand and number of patent LA at the end of EVAR. Significant relationship was observed between aneurysm sac shrink/expand and number of patent LA by ROC analysis (AUC = 0.7556, p = 0.0003). The cutoff value of patent LA number was estimated as 2 by the Youden’s index. ROC: receiver operating characteristic; EVAR: endovascular aneurysm repair; LA: lumbar artery; AUC: area under the curve.

In the patients who underwent coil embolization of aortic branches, the success rate of coil embolization was 43 out of 45 branches (95.6%) for the IMA and 82 out of 111 branches (73.9%) for the LA. In the univariate analysis of patent aortic branches at EVAR completion, the patent IMA and the number of patent LA were significant factors (**Table 1**). Upon performing an ROC analysis of the effect of the number of patent LA on aneurysm shrinkage and expansion, the estimated cutoff value from the Youden’s index was two arteries with an area under the curve of 0.7556 and p = 0.0003 (**Fig. 2**), and in the univariate analysis, less than two patent LAs was also a significant factor (**Table 1**).

With regard to T2-EL, there were patients with T2-EL at 1 week and 6 months after EVAR in the shrinkage group, although the frequency was significantly lower than that in the expansion group, but there were no patients with T2-EL observed at 1 year after surgery in the shrinkage group. In the expansion group, T2-EL was detected frequently even after 1 year (**Table 1**).

A multivariate analysis of patent aortic branches at EVAR completion revealed occluded IMA (odds ratio 7.76) and less than two patent LA were significant factors (odds ratio 3.90) (**Table 2**).

## Discussion

In the mechanism underlying AAA rupture prevention by EVAR, it is thought that thrombi formed by blood trapped between the aneurysm wall and the stentgraft (SG) placed within the AAA become a structure that supports the SG, which excludes the aneurysm from systemic circulation and pressure within the aneurysm becomes lower than systemic pressure.^3)^ Therefore, aortic aneurysm diameter shrinkage following EVAR is considered a predictor of good long-term prognosis, such as high free rate for secondary intervention, EVAR-related complication, and good survival rate.^2)^ As factors influencing on the long-term prognosis of EVAR, endoleak with residual blood flow in the aneurysm sac has a major impact, and HP-EL such as type I and type III, with these endoleaks, intra-aneurysm sac pressure would be high, are known to be risk factors of AAA rupture following EVAR from the time of SG introduction and are thought to require early treatment.^4,5)^ T2-EL is the most frequent after EVAR, and 54%–80% of T2-ELs detected early after EVAR disappear spontaneously. However, persistent T2-EL, which persists for 6 months or more after EVAR, has been thought to be the risk factor of aneurysm sac expansion.^6)^ If blood that remains and stagnates between the SG and aneurysm wall coagulates, blood stagnates within an aortic branch resulted thrombi formation in the aortic branch. Consequently, the aortic side branch is occluded with clot and the T2-EL disappears spontaneously. Therefore, it is thought that the balance of the coagulation and fibrinolytic systems following EVAR influences on T2-EL disappearance or persistence. It has been reported that the coagulation system is activated immediately after EVAR, peaking at days 1–3, and return to the preoperative level 7–10 days after EVAR.^7)^ On the other hand, the fibrinolytic system is activated around 7 days after EVAR, and the activation of fibrinolytic system persists for 3 months.^8)^ Therefore, a coagulation-dominant early after EVAR and relatively coagulation-dominant state caused by activation of the coagulation system or a status due to suppression of the fibrinolytic system from the time of activation of the fibrinolytic system would be beneficial for the spontaneous disappearance of T2-EL.

Furthermore, even persistent T2-ELs, not all T2-ELs, necessarily cause aneurysm sac expansion. The reason why some T2-EL cause sac expansion and others do not would be that the pressure of the cavity within the aneurysm sac with residual blood flow caused by T2-EL (T2-EL cavity) is not constant, and the degree to which this pressure is transmitted to the aneurysm wall through clots would be varied due to the clot quality. In complex IMA–LA type involving the IMA and LA, pressure within the T2-EL cavity is frequently the same as blood pressure,^9)^ and aorta aneurysm expansion has been reported in T2-ELs from aorta branches that have a large inner diameter, and thought to have a high pressure within the T2-EL cavity.^10)^ In a study that measured pressure at various sites within the aneurysm sac after EVAR and evaluated the mean pressure index (MPI) in relation to blood pressure, it was reported that the MPI within thrombi (45%) was lower than the MPI in the T2-EL cavity (79%). Also, the MPI within thrombi in patients with aneurysm sac shrinkage was significantly lower than that in patients with sac expansion or without sac shrinkage.^11)^ The characteristics of thrombi and pressure transmission are significantly related, and when thrombi are organized, pressure is not transmitted.^12)^ Therefore, it is considered highly possible that the intra sac thrombi organization resulted in aneurysm sac shrinkage. Intra sac thrombi would become an organized clot when the coagulation factors’ concentration is high in the trapped blood in the aneurysm sac. Rašiová et al.^13)^ reported that high preoperative fibrinogen levels result in aorta aneurysm sac shrinkage after EVAR, and they speculated that intra sac thrombi form fibrin clots, which contribute to clot stiffness, strength, and stability. With regard to the effect of platelets, Inoue et al.^14)^ classified T2-EL into malignant if the aneurysm sac expanded due to T2-EL or T2-EL resulted in HP-EL occurrence, or benign other than malignant T2-EL and evaluated the platelet count on postoperative day 7. They found that recovery of the platelet count on postoperative day 7 was significantly poor in malignant T2-EL, and the frequency of sac expansion and secondary treatment for sac expansion was significantly high in patients with a platelet count on postoperative day 7 less than 113% of the preoperative level. Based on this result, it is conceivable that recovery of the platelet count on postoperative day 7 could serve as an indicator of aneurysm sac exclusion from systemic circulation.

As medicines that affect the balance of the coagulation and fibrinolytic systems, some patients with AAA take oral antiplatelet agents for arteriosclerosis-related cerebral infarction and ischemic heart disease, or take anticoagulants for atrial fibrillation. These medicines inhibit the coagulation system and might have adverse effect on the long-term outcomes of EVAR. In 2011, we reported that multiple antiplatelet agents and T2-EL inhibit aneurysm sac shrinkage following EVAR,^15)^ and that antiplatelet agents are a factor of aorta aneurysm sac nonregression^14)^ or aorta aneurysm sac expansion.^16)^ With regard to the effect of anticoagulation on the frequency of endoleaks, there are conflicting reports.^17)^ When T2-ELs are observed on angiography at completion of EVAR, it has been reported that T2-ELs significantly persist in patients who received anticoagulation therapy following EVAR, and the effect of which does not differ between warfarin and direct oral coagulation.^17)^ Furthermore, it has also been reported that in patients who received anticoagulation therapy in addition to aspirin, shrinkage of the aorta aneurysm sac diameter was not obtained, and T2-ELs often persist.^18)^ On the basis of these results, it is considered highly possible that anticoagulation therapy inhibits aorta aneurysm sac shrinkage. In our present study, the effect of anticoagulation therapy was not detected. This result might be due to only 10% of patients receiving anticoagulation therapy in both groups.

The state of hyperfibrinolysis following EVAR is thought to persist for several months postoperatively,^8)^ and therefore, following EVAR, it is possible that the coagulation system will become relatively dominant when antifibrinolytic therapy is administered for several months and improve the prognosis of EVAR. TXA is a synthetic derivative of lysine with an antifibrinolytic action by inhibiting the capacity of fibrin to bind to plasminogen,^19)^ and that is a drug that can be taken orally with an antifibrinolytic effect. We administered TXA for 6 months following EVAR with the expectation that blood trapped in an aneurysm sac became thrombi and thrombi became an organized clot.^20)^ As a result, irrespective of taking antiplatelet therapy or not, similar sac shrinkage was obtained and the sac non-shrinkage effect of antiplatelet agents can be neutralized by antifibrinolytic therapy using TXA. However, the frequency of T2-EL after 6 months following EVAR did not decrease with TXA administration: 27% in the group without TXA and 38% in the group with TXA. The frequency of obtaining aneurysm sac shrinkage of 5 mm or more at 6 months after EVAR was significantly higher with TXA administration in patients without T2-EL (80% in the group with TXA administration and 45% in the group without TXA administration, p = 0.0015), and in patients with T2-EL, TXA had no significant impact on the change in sac diameter (22% in the group with TXA administration and 13% in the group without TXA administration, p = 0.410). Therefore, the sac regression effect of antifibrinolytic therapy using TXA alone was insufficient, and the prevention of T2-EL was considered necessary.

Conceivable means to decrease the frequency of T2-EL include either making the coagulation dominant locally within the aneurysm sac or reducing the number of patent aortic branches at the completion of EVAR.

Means of making the coagulation system dominant within the aneurysm sac include placing a coil within the sac during EVAR or intra sac embolization by injecting fibrin glue into the sac after placing a coil into the sac. Li et al.^21)^ conducted a meta-analysis of aneurysm embolization. As a result, they reported that the frequency of T2-EL was significantly lower at 2%–26% (8.4% in average) in patients who underwent intra sac embolization compared to 15%–73% (average 28.4%) in patients who did not undergo intra sac embolization, with no significant difference observed in the rate of complications. They also reported that intra sac embolization during EVAR will highly likely improve long-term prognosis.

Conceivable means to reduce the number of patent aorta branches at the time of EVAR completion include embolization of aortic side branches with a coil or liquid embolic agent before or during EVAR. In a meta-analysis of coil embolization of aortic side branches by Yu et al.,^22)^ it was found that the success rate of coil embolization was 82.3% for the IMA and 69.1% for the LA, and the frequency of T2-EL significantly decreased from 38.6% to 18.5% as a result of coil embolization.

The frequency of T2-EL in EVAR performed with additional procedures, such as intra sac embolization or aorta branch coil embolization, was significantly lower than that of the control group at 8.4% with intra sac embolization^21)^ and 18.5% with coil embolization.^22)^ However, the frequency of T2-EL was even lower at 1.3%–2.2% in EVAR with coil embolization and postoperative antifibrinolytic therapy with TXA,^23,24)^ and it is possible that the frequency of T2-EL would be reduced by intra sac embolization using coil embolization and antifibrinolytic therapy than intra sac embolization or coil embolization alone. In a report examining the frequency of T2-EL according to such coil embolization combined with antifibrinolytic therapy, the frequency of T2-EL was reported to be 27.8% in patients who did not receive either, 29.4% in patients who received coil embolization alone, 24.0% in patients who received antifibrinolytic therapy alone, and 9.8% in patients who received coil embolization with antifibrinolytic therapy, and the frequency of T2-EL was significantly lower in the group who received antifibrinolytic therapy using TXA in addition to coil embolization.^25)^ Therefore, it was suggested that reducing the number of patent aorta branches at the completion of EVAR and administering antifibrinolytic therapy after EVAR will increase the possibility of the prevention of T2-EL. Furthermore, with regard to aorta branch embolization using a liquid embolic agent, it has been reported that the frequency of T2-EL at 1 week after EVAR was reduced to 2.4% by coil embolization of IMA and embolization of the LA using intra sac injection of a liquid mixture of N-butyl-2-cyanoacrylate, which is used off-label in Japan, with ionized oil (lipiodol).^26)^ Based on this report, it is possible that aortic side branch embolization using a liquid embolic agent not only embolized the aorta branches but also has an intra sac embolization effect.

In our present study, when the IMA was occluded and two or less LA were patent at EVAR completion, there was a high possibility of being able to obtain sac shrinkage upon administering antifibrinolytic therapy using TXA following EVAR. Sirignano et al.^27)^ considered T2-EL subject to laparotomy, in which blood inflow into the sac could not be inhibited even after 6 months had passed after endovascular management, to be a cause of aorta aneurysm expansion as “refractory.” In their study, large aneurysm diameter, patent IMA, three or more patent LAs, EVAR without IMA and LA embolization during EVAR, and EVAR without additional intra sac embolization were the risk factors of refractory T2-EL. This result suggested that a good prognosis in EVAR might be obtained when additional procedures are performed that might contribute to the prevention of T2-EL during EVAR, such as occlusion of the IMA, and reduce the number of patent LA to two or less, which is consistent with the sac shrinkage factors that we examined. The success rate of LA embolization during EVAR increases with experience; however, in patients with aortic angulation or in those with a large sac diameter, embolization would be difficult. Problems of coil embolization during EVAR include prolonged operation and fluoroscopy duration, increased radiation exposure, and increased use of contrast medium. When the complex technique required for EVAR, such as shot proximal neck, severely angulated proximal neck, and severely angulated aneurysm or iliac arteries, and when coil embolization are difficult, favorable long-term result might be expected without excessive invasiveness even if terminate of LA coil embolization when patent LA became two or less.

We examined only patient’s background and patent aortic branches at EVAR completion, however, blood pressure might be a factor enabling intervention after EVAR. When blood pressure is low soon after EVAR, T2-EL readily disappears in a spontaneous manner, and when hypertension persists, persistent T2-EL readily occurs and can affect aortic aneurysm sac expansion. It has been reported that if a mean blood pressure is controlled within 75–90 mmHg for 2 days after EVAR, the frequency of T2-EL decreased.^28)^ Also, if the blood pressure before EVAR is controlled at less than 130 mmHg, the frequencies of T2-EL, sac expansion, and secondary treatment were reduced, and the frequency of sac shrinkage was increased if blood pressure is controlled prior to and after EVAR.^29)^ We believe that blood pressure control would be a topic for future study.

Problems of the present study were that antifibrinolytic therapy using TXA, which can contribute to sac regression, and coil embolization during EVAR were introduced during the study period, and the number of patients in the sac expansion group was only 18. Furthermore, in five patients (3%), the aortic aneurysm sac shrunk by 5 mm or more and then subsequently expanded to the preoperative aortic aneurysm diameter or by 5 mm or more compared to the preoperative aortic aneurysm diameter. Accordingly, it is possible that even in patients who were classified into the sac shrinkage group, it is possible that continued follow-up observation would reveal re-expansion, and we believe that even if shrinkage of 5 mm or more is obtained, continued follow-up observation is mandatory. Moreover, we believe that factors that affect re-expansion after shrinkage should be examined. What is more, in the present study, patients with a sac diameter change of less than 5 mm were excluded; however, the purpose of EVAR is to prevent aortic aneurysm rupture, and we believe that the purpose of EVAR can be achieved unless the sac expands. Therefore, we believe that in the future, factors that affect sac expansion should be examined including patients without sac diameter expansion even after long-term follow-up observation of 5–10 years.

## Conclusion

The possibility of significant sac shrinkage would be high when the IMA is occluded; there are two or less patent LAs at EVAR completion, and postoperative antifibrinolytic therapy is performed using TXA.

## Ethics Statement

The present study was approved by the Showa University Research Ethics Review Board (reception number 3125).

## Disclosure Statement

The authors and coauthors have no conflicts of interest to declare.

## Author Contributions

Study conception: AA

Data collection: KM and TM

Data analysis: AA

Investigation: AA, KM, and TM

Manuscript preparation: AA and TO

Critical review and revision: all authors

Final approval of the article: all authors

Accountability for all aspects of the work: all authors.
